# Complex Study of Straw Suitability for the Production of Nonindustrial Straw Pellets

**DOI:** 10.1021/acsomega.3c07057

**Published:** 2023-11-30

**Authors:** Mykola M. Zhovmir, Jaroslav Moško, Josef Farták, Ivo Jiříček, Michael Pohořelý

**Affiliations:** † Institute of Renewable Energy of the National Academy of Sciences of Ukraine, Hnat Khotkevych St. 20A, 02094 Kyiv, Ukraine; ‡ University of Chemistry and Technology, Prague, Technická 5, 16628 Prague 6, Czech Republic; § Institute of Chemical Process Fundamentals, Czech Academy of Sciences, Rozvojová 1, 16500 Prague 6, Czech Republic

## Abstract

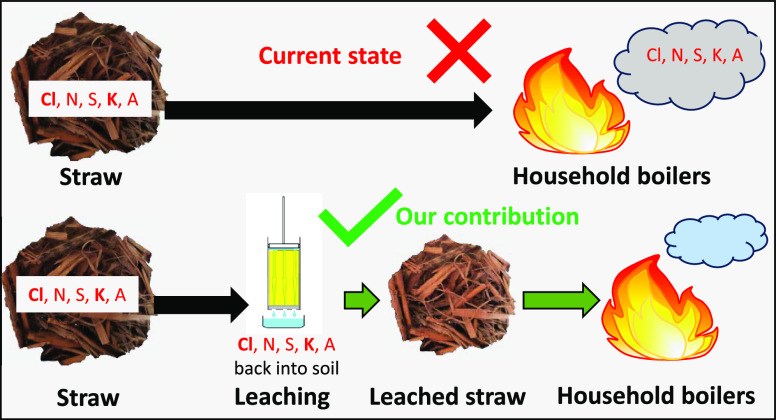

Nonindustrial straw pellets should comply with limitations on the content of ash, chlorine, nitrogen, sulfur, and heavy metals, and have a high melting temperature of ash. To produce such pellets, the properties of straw can be improved by leaching. In known papers, the completion of chlorine washing-out was not controlled. Aims of the paper were to study ash solubility at leaching of straw until completion of chlorine removal and to make a conclusion on studied straw suitability for the production of nonindustrial pellets. Aims were achieved by straw soaking with heating to 100 °C and subsequent plug flow flashing with control of leaching completion by the absence of chlorine in leachate; studying the ash, chlorine, nitrogen, sulfur, and heavy metals content of straw; studying the thermal behavior of ash at heating; determining the initial deformation temperature (IDT) of ash; and comparing the properties of original and leached straw with the specification of straw pellets. Straw leaching until completion of chlorine washing-out provided decreasing chlorine, nitrogen, and sulfur contents below limitations, and the ash content decreased from 7.15 to 3.93% at water leaching to 4.29% at leaching with a 10% solution of acetic acid. In the ternary diagram, the composition of straw ash shifted from a zone of low melting eutectics to zones of high-melting tridymite and cristobalite. The IDT of the original straw ash was 847, 1250 °C after water leaching, and above 1275 °C after leaching with an acetic acid solution. Monitoring the absence of chlorine in the leaching liquid can be applied as a control parameter for straw leaching completion. The original straw was not suitable for the production of nonindustrial pellets because of the high contents of Cl, S, and Cr and the low IDT of ash. All indexes of straw were improved due to leaching, but the Cr content was above limitation. Producers of pellets need to assess straw suitability as to heavy metal content both in the original and leached states.

## Introduction

The current task of energy development is to increase renewable energy usage, particularly biomass. By joining the European Energy Community, Ukraine accepted commitments to achieve an essential share of renewable energy in the final energy consumption. The Energy Strategy of Ukraine for the period until the year 2035 envisaged an increased contribution of renewable energy up to 25%, and the contribution of biomass should reach 11 million metric tons of oil equivalent (m toe) in 2035.^1^

In Ukraine, technically achievable solid biomass resources for energy need to make up to 35 m toe annually, enough to achieve biomass energy goals. At the current level of economic activity, about 2.7 m toe of firewood and wood waste can be technically available for energy needs and 10.9 m toe of cereal straw.^2^ Wood resources in general are restricted, and with the transition to a circulating economy, the availability of wood, especially round wood, for energy needs will decrease. At limited resources of wood fuels to increase biomass contribution to the energy balance, there is a necessity for the widespread usage of cereal straw as fuel.

According to our estimates, 53 boilers with periodic burning of whole straw bales were operated in Ukraine before 2022; their total heat output was 31 MW, and their yearly consumption of baled straw was only near 25,000 metric tons. Factors such as unfavorable logistics of straw, high capital costs of boiler houses with the burning of whole straw bales, and the inability to build straw warehouses and boiler houses in most towns constrain their application.

Overcoming these obstacles is possible by producing straw pellets and using them as fuel. Pellets are more convenient and safer for transportation and storage, allowing the complete mechanization and automation of their usage. In Ukraine, several straw pellet factories have been built, mainly to export straw pellets as fodder and litter for animals. Wood pellet production and burning are currently mastered technologies.^3^ Nevertheless, the direct application of wood pellet technologies for the production and burning of straw pellets currently is impossible.

All pellets can be classified according to ISO 17225-1:2017.^4^ Pellets according to this standard can be produced from biomass in the same condition as those delivered from fields and forests. This standard establishes the normative and reference characteristics of pellets. For standardized technical characteristics, gradations of their numerical values are provided: nominal diameter and length of pellets, moisture content, ash content, mechanical strength, fines content, additives used in production, bulk density, and the minimum value of the low calorific value in working conditions. Reference characteristics of pellets with gradations of numerical values are also given: the content of nitrogen, sulfur, and chlorine and data on the pellet’s length distribution. As a reference, data on the actual values of the temperature characteristics of ash melting behavior should be given.^4^ Based on these data, it is possible to determine the possible directions of industrial or nonindustrial use of individual batches of pellets with homogeneous characteristics.

Straw pellets can be regarded as a promising fuel for boiler houses in municipal heat supply and for domestic boilers in the Steppe Zone with intensive grain cultivation and limited wood fuel resources. Boiler houses of decentralized municipal heat supply and small domestic and commercial heating boilers are regarded as nonindustrial consumers. Often, such boilers are placed in densely populated districts, operated with simple burning technology and control systems, as a rule without or with the simplest flue gas cleaning, by low-qualification personnel or household owners. That is why nonindustrial straw pellets should comply with strict specifications stated in ISO 17225-6:2014.^5^ This standard envisages pellet production from grass biomasses and biomass mixtures, including straw, or completely from cereal straw.

Cereal straw, as a raw material for pellet production, has varying contents of chlorine, nitrogen, and sulfur according to data presented in ISO 17225-1:2017^4^ and limitations to ash, Cl, N, and S contents in nonindustrial straw pellets were categorized and established by ISO 17225-6:2014.^5^ Comparing the typical Cl content of cereal straw with limitations for its contents of straw pellets (Table 1), it is clear that direct usage of native straw from fields for production of nonindustrial straw pellets is practically impossible, whereas the N and S content of straw is not so critical. Besides ash, Cl, N, and S contents, there are restrictions on the content of heavy metals such as As, Cd, Cr, Cu, Pb, Hg, Ni, and Zn, whose content in natural straw can sometimes surpass allowable limits.

**Table 1:** Composition of Cereal Straw and Limitations for Nonindustrial Pellets

parameters	cereal straw, ISO 17225-1^4^		cereal straw pellets, ISO 17225-6^5^	grass pellets, ISO 17225-6^5^	
	typical content	possible range		class A	class B
ash, [wt %]	5	2–10	for class A6.0: < 6.0; for class A6.0+: > 6.0	for class A6.0: < 6.0	for class A10: < 10
N, [wt %]	0.5	0.2–1.5	N0.7 ≤ 0.7	N1.5 ≤ 1.5	N2.0 ≤ 2.0
S, [wt %]	0.1	0.05–0.20	S0.10 ≤ 0.10	S0.20 ≤ 0.20	S0.30 ≤ 0.30
Cl, [wt %]	0.4	0.1–1.2	Cl0.10 ≤ 0.10	Cl0.10 ≤ 0.10	Cl0.30 ≤ 0.30
As, [mg/kg]	<0.1	0.1–2.0	≤1	≤1	≤1
Cd, [mg/kg]	0.10	0.05–0.30	≤0.5	≤0.5	≤0.5
Cr, [mg/kg]	10	1–60	≤50	≤50	≤50
Cu, [mg/kg]	2	1–10	≤20	≤20	≤20
Pb, [mg/kg]	0.5	0.1–3.0	≤10	≤10	≤10
Hg, [mg/kg]	0.02	0.02–0.05	≤0.1	≤0.1	≤0.1
Ni, [mg/kg]	1	0.2–4	≤10	≤10	≤10
Zn, [mg/kg]	10	3–60	≤100	≤100	≤100

The presence of Cl, N, and S in solid biofuels, including straw pellets, influences fuel usage. At burning, they cause pollutant emissions such as NO_*x*_, SO_2_, SO_3_, and HCl. Increased emissions of chlorinated aromatic compounds such as dioxins are associated with the presence of Cl in fuel. The formation of these pollutants also depends on the parameters of burners, furnaces, and operation conditions, but higher concentrations of Cl, N, and S in fuel cause an increase in the formation of the named pollutants.^6^ At straw burning, even in medium 0.6–9 MW boilers, there were the following average emissions 1200 mg/m^3^ of CO, 180 mg/m^3^ of NO_*x*_, 260 mg/m^3^ of SO_2_, 80 mg/m^3^ of HCl, up 200 mg/m^3^ of particles, from 0.8 × 10^–6^ to 0.8 × 10^–6^ mg/m^3^ of dioxins (PCDD + PCDF), some of them were above allowable limitations.^7^ Thus, the use of straw pellets with limited N, S, and Cl content that meet the standard ISO 17225-6:2014^5^ in small boilers will contribute to the compliance of heating boiler operation with current environmental restrictions on pollutant emissions.

Compared to wood, the cereal straw has an increased ash content with higher content of alkali metals and chlorine salts.^4^ At straw burning, segregation of inorganic elements between bottom and fly ash occurs. The high concentrations of K, Na, Cl, and S in fly ash are of great relevance for reactions that can take place in the boiler section where the flue gas is subjected to a considerable temperature gradient, which is accompanied by chemical reactions, phase transitions, and precipitation processes that can support or initiate fouling and corrosion.^8^ Grate and wall deposits were similar in composition to that of the fuel ash. Potassium and calcium silicates and sulfates were deposited on screen tubes and superheaters. Chlorides and carbonates appeared in the cooler convection passes. Fireside deposits and convection pass fouling reduced the plant availability and efficiencies.^9^ Chlorine is a major factor in deposit formation. Chlorine facilitates the mobility of many inorganic compounds, in particular potassium. Chlorine concentration often dictates the amount of alkali vaporized during combustion more strongly than the alkali concentration in the fuel.^10^ Studying the binary alkali–silica phase diagram showed the lower liquidus temperatures for sodium-silicate and potassium-silicate from 40 to 100% silica concentration, and especially low at alkali content from 20 to 40%.^11^ Low-temperature melting of straw ash can lead to bed agglomeration and slagging at the boiler grate. At straw pellet combustion in small heating boilers, resulting melt ash clusters resulted in blocking combustion with high CO emissions and led to a sharp drop in heat output and burning extinction.^12^

Ash melting behavior of biomass ash should be determined according to ISO 21404:2020 with measurement of the following temperatures: shrinkage starting temperature, SST; deformation temperature, DT; hemisphere temperature, HT; and flow temperature, FT.^13^ Often, the melting behavior of biomass ash is studied according to the American standard method ASTM D1857, which involves determining the initial deformation temperature, IT (often abbreviated as IDT); softening temperature, ST (often abbreviated as SOT); hemispherical temperature, HT; and fluid temperature, FT.^14^ From comparing the standards mentioned, there are differences in the form of the test piece of ash and in characteristic signs of melting stages. That is why some disagreements in the data from different papers can be found.

Based on data on ash melting behavior for 24 samples of wheat and barley straw, the ranges were found for IDT 720–1120 °C, SOT 760–1110 °C, HT 1038–1280 °C, and FT 1080–1500 °C,^15^ which were determined in oxidative conditions according to ASTM D1857. According to data^16^ obtained based on a study of 51 straw ash samples, the SOT ranged from 775 to 1225 °C, with the most likely value of 925 °C. According to the study of 5 samples, the SOT of wheat straw ash was 726–840 °C.^17^

The burning of straw pellets in retort burners and burners with movable grates, those commonly applied for wood pellets, was complicated by ash agglomeration, disruption of their work with a significant decrease in heat output and reduced energy efficiency, and increased CO emission.^18^

According to ISO 17225-6:2014,^5^ the nonindustrial straw pellets should be suitable for combustion with burners complying to EN 15270:2007,^19^ in boilers running on pelletized fuel and meeting the requirements of EN 303-5:2012.^20^ Although ISO 17225-6:2014^5^ does not specify requirements for the melting temperature characteristics of straw pellet ash, such requirements can be derived from the requirements of the “ENplus pellets certification system”, stating ash deformation temperatures (DT) for wood pellets of class A1 above 1200 °C and for A2 and B classes above 1100 °C.^21^ At that, DT should be determined according to CEN/TS 15370-1:2006^22^ which was substituted with ISO 21404:2020.^13^ This means that the properties of straw for nonindustrial pellet production should be modified to increase the temperature characteristics of ash melting to achieve at least DT > 1100 °C. Guided by an aim to ensure ash deformation temperature of straw pellets above 1100 °C, the straw pellet’s producer can gain a wide market with huge consumption and premium prices, giving coverage of expenditures for additional processing of straw.

There are different approaches for improving the properties of straw as fuel, first of all to raise temperatures characterizing ash melting, among them: straw weathering in the field with partial removal of chlorine and alkali metals by rainwater and dew;^7^ leaching of straw with water giving removal of chlorine and water-soluble part of alkali metals, improving combustion^23^ and gasification^24^ of straw; and leaching with water solutions of different acids (acetic acid,^25^ sulfuric acid,^26^ and carbonic acid^27^) allowing the removal of alkali and alkali-earth metals by leaching and ion exchange. Application of additives to straw^28^ or straw pellets^29^ to raise ash melting temperatures was also studied, which is not the subject of this paper.

According to a review of Staniforth, as far back as the 1950s, it was known that delayed grain harvesting in the presence of rain and dew resulted in a decrease in the ash content of straw. It was practiced purposefully to leave threshed straw in the field to somewhat reduce the ash content through natural washing with rain and dew.^30^

It was found that with the accumulation of precipitation to 100 mm, the chlorine content in straw decreased sharply from 0.5 to values less than 0.1% and potassium from 1.2 to 0.2%, but further precipitation almost did not lead to a significant decrease in their content.^31^ With the accumulation of precipitation up to 100 mm, the content of nitrogen in straw decreased by 40%, sulfur by 30%, chlorine by 78%, and potassium by 46%. The practical implementation of straw washing by rain requires considerable time and land area and in almost 100 days, the chlorine content in straw decreased from 0.8 to 0.4%, potassium from 1.8 to 0.8%, and nitrogen from 1.3 to 0.6%.^32^ Such weathered straw acquired a gray color and is characterized by reduced chlorine content to 0.2 wt % and ash to 3 wt %, and ash SOTs were in range of 950–1100 °C.^7^ So, naturally occurring leaching cannot be regarded as acceptable and sufficient to improve straw quality for the production of nonindustrial pellets. In addition, the need to free fields for further agricultural work, especially in the dry climate of the Steppe zone, makes a limitation for straw weathering with rain and has prompted interest in the artificial washing of straw.

It was found easy to remove potassium, sodium, and chlorine from wheat straw at water leaching, with total ash reducing up to 68%. The processes of wheat straw leaching, applying approaches of spraying water over straw beds and soaking straw samples with the following flushing water through them, were studied, soaking being more effective in removing alkali metals and chlorine. Straw leaching was controlled via measurement of the electrical conductivity of leachate. To complete leaching, the application of 0.04 L/g water was sufficient, which is equivalent to 24 mm of precipitation.^23^

Jenkins et al. analyzed the binary phase diagram for alkali oxides Na_2_O and K_2_O with silica SiO_2_ and found that the melting temperature for high alkaline ash of wheat straw can even decrease if insufficient leaching occurs to shift the composition above about 85% silica. For wheat straw, having ash with an initial silica content of 50% partial leaching with increasing ash silica content to 58% leads to melting temperature decreasing from 980 °C to below 800 °C; progressive leaching with increasing silica content in ash from 58 to 78% resulted in parabolic changing of ash melting temperature having a local maximum of 1040 °C at silica content of 68%, and near 760 °C at 78% silica content. The increase in melting temperature of ash is stable with a silica content above 80%.^11^

Many researchers carried out straw leaching at different temperatures, from 25^33^ up to 100 °C.^34^ Consumption of distilled, deionized or tap water for straw leaching was characterized by water-to-straw ratio, which was from (0.00525–0.024) L/g (Thompson et al.),^33^ 0.012 L/g (Wu et al.),^35^ 0.040 L/g (Jenkins et al.),^23^ 0.050 L/g (Ma et al.),^36^ and up to 0.065 L/g (Alabdrabalameer et al.).^24^ Straw leaching was often conducted without control of leaching completion; measurements of leachate electrical conductivity or water consumption were not reported.^37,38^

Incomplete leaching may be indicated by a high content of residual chlorine in the ash of leached straw, for example, 2.2%, as in paper.^37^ Application of a low water-to-straw ratio can lead to incomplete leaching and may be justified by the low content of chlorine in the ash of original straw as in the paper,^35^ and leaching with excessive spending of fresh water cannot be as acceptable as giving polluted water (sewage), which should be disposed of or purified.

Sequential leaching of biomass with water and aqueous solutions of organic and mineral acids is used to characterize the binding of ash-related elements with fuel. Ash-related elements such as sulfates, phosphates, and alkali metal chlorides are washed out with water. It was believed that an aqueous solution of ammonium acetate NH_4_Ac washes cations Mg, Ca, K, and Na, which are bonded to the organic matter of the fuel. Hydrochloric acid solution washes out alkaline-earth carbonates, sulfates, and other metals. It was supposed that silicates and other minerals would remain in the insoluble residue.^39^ Heavy metals are leached at low pH values except Zn, Pb, and Mn, which may be present in fuels in water-soluble and/or ion exchange forms.^40^ In the residual fraction of leaching, K and Na were found and regarded as components of mineral soil contamination of fuel.^25^ It can be assumed that depending on the composition of the ash-related elements of the original straw and the requirements for solid biofuels planned to be produced from it, it may be necessary to use one or a combination of leaching agents, and not all undesirable components can be leached completely.

There are different approaches to describing the behavior of leached straw ash: measurements of ash composition with calculations changing slagging and fouling indexes,^38^ describing the behavior of leached straw ash in energy installation,^24^ and determining characteristic temperatures of ash melting behavior.^35^ In the last-mentioned paper, it was shown that due to appropriate water leaching, the ash deformation temperature increased from 920 °C for the original straw to 1320 °C for the leached, and for another sample of straw, from 910 to 1250 °C.^35^ In study^41^ for the sample of original wheat straw, the ash deformation temperature was 1020 °C, and for the ash of water-leached straw it increased only to 1050 °C.

In the considered papers, the completion of straw leaching was not always controlled by measuring the electrical conductivity of the leachate. Although the presence of chlorine in the straw and in its ash is considered undesirable, the removal of chlorine with washing water was not controlled in studies. The presence of chlorine in the chemical composition of the ashes of the original and leached straw was sometimes not reflected. The published data describe the identified dependencies and demonstrate changes in the ash properties of these studied straw samples. To answer the question about the suitability of the straw available in fields for the production of straw pellets, especially for nonindustrial usage, it is necessary to study the properties of this particular straw and its ash, as well as the achievable changes in properties at the application of different technologies of straw leaching.

The objectives of the paper are to study the solubility of wheat straw components at straw leaching with water or with a water solution of acetic acid until completion of chlorine removal; to determine the chemical composition of insoluble and soluble ash of straw; to estimate heavy metal content in original and leached straw; to characterize the changes in the behavior of ash during heating that are caused by straw leaching; to determine the IDT of ash of the original straw and straw after leaching, based on the found data to make a conclusion on the suitability of studied straw for pellet production, especially for pellets for nonindustrial usage.

## Materials and Methods

### Experimental Approach

#### Straw for Experimental Research

Straw of winter wheat from a field near the town of Liběchov (Central Bohemian Region of the Czech Republic) harvested in 2021 was used for research. The preparation of straw for research and the selection of analytical samples were carried out according to requirements of standard ISO 14780:2017.^42^ Straw was cut into particles up to 30 mm long with scissors and then ground in a mill to particles less than 0.7 mm. The straw sample before grinding, as well as ground straw, was weighed on scales with a resolution of 1 g, and the material balance failure was less than 2% of the initial sample weight because of heating in the mill with partial drying. Ground straw was thoroughly mixed, and analytical samples of the original straw weighing 100 g each were isolated. Test portions weighing about 5 g were taken from the analytical sample for determining the moisture *W*_or_ and ash content A_d_ of the original straw and for leaching experiments.

#### Straw Leaching with Distilled Water

A test portion of original straw, crushed to particles less than 0.7 mm, having a weight *m*_or_ of about 5 g, was taken and weighed on scales with a resolution of 0.001 g. Leaching was carried out in two stages: soaking and subsequent plug flow flushing. The test portion of straw was placed in a 500 mL flask, and 100 mL of distilled water, characterized by a residual salt content of about 1 ppm and an electrical conductivity of 3 μS/cm, was added. The straw particles were slowly soaked with water, and even after stirring, about half of the particles remained floating on the surface of the water. Therefore, the accelerated method of leaching with heating^43^ was applied. The flask was placed in a sand bath, heated, and boiled on low heat for 20 min. After the heating stopped, almost all the straw particles were soaked and sank to the bottom of the flask. It was assumed that air was removed from the capillaries and water penetrated into them, ensuring the dissolution and leaching of salts.

**Figure 1:**
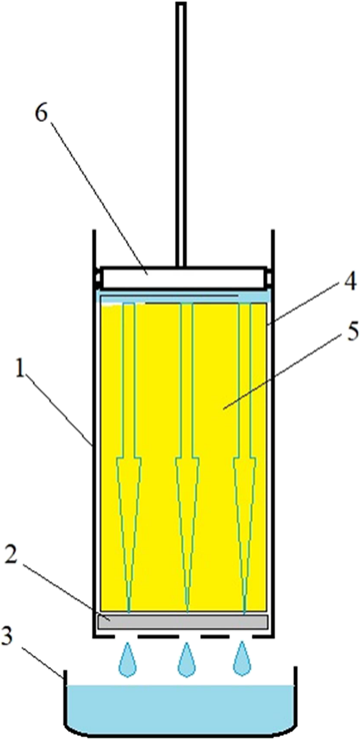
Scheme of the hand press for plug flow leaching of straw: (1) cylinder, (2) porous felt insert, (3) pot, (4) fabric filtering bag, (5) straw, and (6) piston.

Separation of straw from leachate was carried out using a hand press (Figure 1). Cylinder 1 had an inner diameter of 34 mm and a height of 135 mm, and the bottom of the cylinder had holes of 4 mm in diameter. A porous felt insert 2 with a 3 mm thickness was inserted at the bottom of the cylinder, and a fabric filtering bag 4 was placed into the cylinder. After being cooled to room temperature, the contents of the flask with straw and extract were poured into a filtering bag. After the liquid from the filtering bag was drained into pot 3, the flask was rinsed with a filtered extract to completely transfer the straw particles to the filtering bag. Then the filtering bag was closed, and piston 6 was inserted into the cylinder and manually pressed on it to squeeze the extract from straw 5 into pot 3. The approximate pressure created by the piston was 0.1 MPa.

The piston 6 was removed from the press, and the filtering bag 4 was opened. The flask, free of straw particles, was rinsed with 15 mL of distilled water, and the flush was poured into a filtering bag 4. Then the filtering bag was closed, and the piston was inserted and pressed on, pushing water through a layer of straw. The piston was removed again, and 15 mL of distilled water was poured onto the filtering bag; a piston was inserted, and water was pushed through a layer of straw to wash out the remaining leachate. Upon completion of the liquid outlet, 2 drops of the extract were taken from the outlet of the press onto a clock glass and tested for the presence of chlorine by reaction with a drop of 1.7 wt % AgNO_3_ (grade: pure for analysis) solution in distilled water. If, after washing with the next portion of distilled water, there was no turbidity at testing with AgNO_3_ solution, then the washing-out of chlorine was considered complete.

The filtering bag with compressed leached straw was kneaded and then evenly distributed over the volume of the tied bag. A bag with leached straw was hung in a warm, ventilated room for drying to a constant mass, *m*_l_. The straw was wiped through a sieve having 1 mm meshes, mixed thoroughly, and placed for storage in a tight container, from where test portions of dried leached straw were taken to determine the moisture content *W*_wl_ and ash content A_dw_. Based on the data obtained, the efficiency of straw leaching with distilled water was determined:
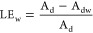
1

where: A_d_ is the ash content of the original straw, wt %; A_dw_ is the ash content of the water-leached straw, wt %.

Yield of dry straw leached with water Yi_w_ was calculated as the ratio of dry weight of leached straw to dry weight of original straw taken for experiment:
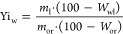
2

where: *m*_l_ is the weight of the air-dry water-leached straw, g; *m*_or_ is the weight of the original straw, g; *W*_wl_ is the moisture content of the air-dry water-leached straw, wt %; and *W*_or_ is the moisture content of the original straw, wt %.

The extract obtained from water leaching of straw was evaporated at room temperature, dried at 105 °C, and used to determine the chemical composition of its mineral part.

Straw leaching with a 10% aqueous solution of acetic acid was carried out according to a method similar to that of washing with water, at which, chemically pure 99.9% glacial acetic acid and distilled water were used for solution preparation. After completion of the chlorine washing-out with acetic acid solution, the final washing was performed with 20 mL of distilled water.

Straw leached with acetic acid solution was dried, wiped through a sieve, mixed thoroughly, and stored in a closed container, from where test portions were taken to determine the moisture *W*_acl_ and residual ash A_da_ content. Based on the data obtained, the efficiency of ash leaching from straw with an aqueous solution of acetic acid LE_acl_ and yield of dry straw leached with solution of acetic acid Yi_acl_ were calculated according to equations similar to eqs 1 and 2.

The extract obtained from the straw leaching with a solution of acetic acid was evaporated at room temperature, dried at 105 °C, and used for determining the chemical composition of its mineral part.

#### Determining Moisture and Total Ash Content

Test portions of 1–2 g were taken from analytical samples of the original straw or dried leached straw for determining moisture and ash content**s** according to standard methods ISO 18134-2:2017^44^ and ISO 18122:2022.^45^ In standard ISO 18122:2022,^45^ to ensure complete carbon burnout, moistening the ash and again burning it out at 550 °C is recommended, but according to the precautions of standards CEN/TS 15370-1:2006^22^ and ISO 21404:2020,^13^ such approach is prohibited, since it can lead to the leaching of soluble components, changes in the chemical composition, and in the temperature characteristics of ash melting. To ensure the carbon burnout from the formed ash, the air, humidified by bubbling through a flask with distilled water heated to 40 °C, was supplied at a rate of 0.07 L/s into the muffle furnace. The ash obtained in parallel experiments was stored for further measurements of chemical composition and characteristic temperatures of ash melting.

Chemical composition of ash-related components present in straw samples, dried leachate, and ash was determined by X-ray fluorescence analysis (XRFA) using a “PANalytical XRF Platform-Zetium” analyzer intended for measurements from Be (*z* = 4) to Am (*z* = 95). Ash-related chemical elements can be found in the form of organic and mineral compounds of various compositions in straw, ash, and leachate. XRFA determined the relative mass content of chlorine Cl, sulfur S, phosphorus P, silicon Si, iron Fe, aluminum Al, calcium Ca, magnesium Mg, sodium Na, potassium K, titanium Ti, and also identified trace elements (Mn, Cu, Zn, Sr, Br, Rb, Cd, and Ba). Conventionally, the composition of the mineral part of straw and of straw ashes was expressed by the relative mass fractions of its components: chlorine Cl and oxides SO_3_, P_2_O_5_, SiO_2_, Fe_2_O_3_, Al_2_O_3_, CaO, MgO, Na_2_O, and K_2_O with normalization to 100%. The content of TiO_2_ was low, up to 0.067 wt %, and therefore data on its content are not given

The content of chlorine and sulfur in solid biofuels should be measured according to ISO 16994:2016, and this standard allows the XRFA method with procedures appropriate for biofuels.^46^ Contents of chlorine, sulfur, and heavy metals (As, Cd, Cr, Cu, Pb, Hg, Ni, and Zn) in samples of original and leached straw were measured by means of an “ElvaX Plus” XRF analyzer (Elvatech, Ukraine) intended for quantitative measurements from Na (*z* = 11) to U (*z* = 92). Total nitrogen content in samples of original and leached straw was determined by the Kjeldahl method according to ISO 5983:2005,^47^ which is commonly used in agriculture for fertilizer and fodder analysis.

Temperature characteristics of ash melting behavior were determined close to ASTM D1857,^14^ with visual observation of changes in the shape of the ash pyramids. Straw ash was ground in a mortar to form particles passing through a 50 μm sieve. Test ash pyramids were 19 mm in height and 6 mm in width at each side of the base, which is an equilateral triangle. To form pyramids, the ash of original straw was moistened with 95% ethyl alcohol and the ash of leached straw with dextrin solution. In the muffle furnace, an oxidizing atmosphere was maintained due to air access through the ventilation holes. To measure the temperature, a K-type thermocouple was used, the tip of which was at the level of the pyramid apex at a distance of 10–15 mm along the axis of the furnace. To indicate temperature, the microprocessor temperature controller RT-0102 (JSC “Lvivprylad”, Ukraine) with readability 1 °C and measurement error ±3 °C was used. While testing, the temperature was increased at a rate of 11–8 °C/min to a temperature of 800 °C and then at a rate of about 8–6 °C/min. The maximum temperature possible to reach in the muffle furnace was 1275 °C. Tests were repeated at least three times for each ash sample.

#### Thermogravimetric Analysis

The behavior of the ash samples at heating was studied by simultaneous thermogravimetric analysis (TGA) and differential scanning calorimetry (DSC). These analyses were performed on a horizontal thermobalance scale SDT Q600 (TA Instruments). At that, approximately 7 mg of sample was heated from room temperature to the target temperature of 1100 °C in air atmospheres with a flow rate of 100 mL/min at a heating rate 10 °C/min. The specified heating rate is close to the recommended heating rate for determining the temperature characteristics of ash melting according to the standards ISO 21404:2020^13^ and ASTM D1857.^14^

### Analytical Approach

The ternary diagram, as was recommended by Lachman et al.,^48^ was constructed taking into account only the most abundant oxides of ash. Following this approach, minor components such as chlorine, SO_3_ and TiO_2_, were omitted, and abundant oxides were split into three groups. The two dolomitic compounds CaO and MgO were grouped together, SiO_2_ was grouped with the other two metallic oxides Al_2_O_3_ and Fe_2_O_3_, and the alkali compounds K_2_O and Na_2_O were grouped with P_2_O_5_. Obtained data on the composition of ash-related components in samples of original and leached straw and the chemical compositions of ashes were plotted into a ternary diagram. Approach proposed in paper^28^ was used for mapping changes of chemical composition of ash in the ternary diagram.

#### Slagging and Fouling Indices

For obtained ashes of original and leached straw, the slagging and fouling indices were determined, and the obtained values were compared with their critical values according to approaches systematically presented in the scientific papers, particularly: silica content (SiO_2_);^59^ chlorine content (Cl);^49^ basic to acidic compounds ratio B/A = (Fe_2_O_3_ + CaO + MgO + Na_2_O + K_2_O + P_2_O_5_)/(SiO_2_ + Al_2_O_3_ + TiO_2_);^48^ bed agglomeration index BAI = (Fe_2_O_3_)/(Na_2_O + K_2_O);^48^ fouling index Fu = (B/A) (Na_2_O + K_2_O);^48^ and slag viscosity index Sr = SiO_2_*100/(SiO_2_ + Fe_2_O_3_ + CaO + MgO),^48^ IDT.^59^

## Results and Discussion

### Results of Straw Leaching

The overall liquid-to-straw ratio in straw leaching with water until chlorine removal was 0.029 L/g. The electrical conductivity for the first portions of the extract was (1670–1700) μS/cm, and for the last portion of washing water in the absence of chlorine, it was (820–880) μS/cm.

The overall liquid-to-straw ratio in straw leaching with a 10 wt % solution of acetic acid until chlorine removal was 0.031 L/g. At completion of chlorine washing-out from straw with acetic acid solution, the leachate acidity was pH = 5 and the electrical conductivity was 2630 μS/cm; the electrical conductivity of the last portion of washing water was 1030 μS/cm.

Data on the content of chlorine, nitrogen, sulfur, and ash in original and leached straw, as well as the efficiency of ash leaching, are given in Table 2. At leaching, the content of chlorine decreased from 0.216 wt % in the original straw to the residual content of 0.038 wt % in water-leached straw, and 0.058 wt % in straw leached with acetic acid solution, respectively. Complete removal of chlorine was not achieved, although chlorine was no longer detected in the leachate. Sulfur content of the original straw was 0.103 wt % and decreased to 0.040 wt % after water leaching and 0.073 wt % after leaching with acetic acid solution, respectively.

**Table 2:** Chlorine, Nitrogen, Sulfur, and Ash Content in Original and Leached Straw, Efficiency of Ash Leaching, and Yield of Leached Straw

indexes	original straw	straw leached with distilled water	straw leached with 10% solution of acetic acid
chlorine content, Cl, [wt %]	0.216	0.038	0.058
nitrogen content, N_d_, [wt %]	0.526	0.386	0.387
sulfur content, S_d_, [wt %]	0.103	0.040	0.073
ash content, A_d_, [wt %]	7.15	3.93	4.29
efficiency of ash leaching, LE, [wt %]		45.1	40.0
yield of dry leached straw as portion of dry original straw, Yi, [wt %]		91.7	94.8

The permissible chlorine and sulfur contents of nonindustrial straw pellets are 0.10 wt % (ISO 17225-6:2014^5^), and therefore as to chlorine and sulfur content, the original straw was unsuitable, but both kinds of leached straw became suitable for the production of the nonindustrial straw pellets intended for heating residential buildings and similar consumers. At straw leaching with acetic acid solution, chlorine and sulfur removal was less complete than in the case of water leaching.

Due to leaching, the nitrogen content decreased from 0.526 near to 0.39 wt % at leaching with both water and acetic acid solutions. The original straw had nitrogen content below the limitation of 0.7 wt %,^5^ and leaching further decreased this index.

Ash of the original straw was gray, which in our opinion was caused by the color of phosphorus salts; the ash of water-leached straw was white with a slightly gray shade, and the ash of straw leached with acetic acid solution was white. Straw leaching until the completion of chloride removal provided a reduction in the ash content. The lower ash content was reached during water leaching.

The efficiency of ash removal is higher at water leaching (45.2%) than at leaching with a 10 wt % solution of acetic acid (40.0%). In terms of the ash content, the original straw was suitable for the production of A6.0+ class pellets with an allowable ash content of more than 6.0 wt %. The leached straw became suitable for the production of higher-quality A6.0 pellets, for which the ash content should be less than 6.0 wt % per dry weight (ISO 17225-6:2014^5^).

Data on the mineral part composition of straw samples, ash, and dried leachates are presented in Table 3. According to data on the composition of the mineral part in the original straw (column 2), in straw leached with water (column 4), and in straw leached with 10% acetic acid solution (column 7), the leaching with water is more effective in removing chlorine and sulfur-containing compounds from straw compared to acetic acid solution leaching. The S content (expressed as oxide SO_3_) in the water-leached straw is less than that of the original straw and acetic acid solution-leached straw, but after burning, the SO_3_ content of the ash of the water-leached straw was higher than in the ash of the acetic acid solution-leached straw. This can be attributed to more SO_3_ binding by the water-leached straw ash, which is richer in the content of K, Ca, and Mg oxides than the acetic acid solution-leached straw ash. Therefore, it is possible to predict a lower emission of sulfur oxides in the flue gases at its burning.

**Table 3:** Composition of Ash-Related Components in Straw and Its Ash

	original straw		straw leached with distilled water			straw leached with 10% acetic acid		
main ash components	mineral part in original straw^a^	ash of original straw^a^	mineral part in water-leached straw^a^	mineral part in dried water leachate^b^	ash of water-leached straw^b^	mineral part in straw, leached with 10% acetic acid^a^	mineral part in dried acetic leachate^b^	ash of straw leached with 10% acetic acid^b^
1	2	3	4	5	6	7	8	9
Cl, [wt %]	4.23 ± 0.04	4.26 ± 0.10	0.79 ± 0.06	4.54 ± 0.03	0.09 ± 0.009	1.09 ± 0.31	3.97 ± 0.03	0.47 ± 0.007
SO_3_, [wt %]	4.25 ± 0.06	5.73 ± 0.17	2.54 ± 0.08	5.75 ± 0.04	1.44 ± 0.04	4.42 ± 0.13	4.68 ± 0.04	0.06 ± 0.02
P_2_O_5_, [wt %]	1.66 ± 0.03	2.67 ± 0.08	1.12 ± 0.005	3.07 ± 0.03	1.07 ± 0.03	1.77 ± 0.05	2.80 ± 0.03	1.10 ± 0.03
SiO_2_, [wt %]	40.42 ± 1.02	50.24 ± 0.2	68.83 ± 0.42	14.18 ± 0.06	81.46 ± 0.1	84.72 ± 0.79	0.85 ± 0.02	94.28 ± 0.1
Fe_2_O_3_, [wt %]	0.44 ± 0.09	0.17 ± 0.02	0.54 ± 0.003	0.22 ± 0.007	0.23 ± 0.01	0.44 ± 0.01	0.32 ± 0.009	0.19 ± 0.01
Al_2_O_3_, [wt %]	0.16 ± 0.004	0.22 ± 0.02	0.30 ± 0.05	0.09 ± 0.05	0.24 ± 0.01	0.28 ± 0.03	0.06 ± 0.004	0.23 ± 0.01
CaO, [wt %]	13.23 ± 0.44	7.07 ± 0.12	13.85 ± 0.03	7.81 ± 0.04	6.58 ± 0.07	3.98 ± 0.02	19.07 ± 0.07	1.76 ± 0.04
MgO, [wt %]	0.79 ± 0.03	1.74 ± 0.06	1.05 ± 0.02	2.04 ± 0.02	1.72 ± 0.04	0.13 ± 0.03	4.09 ± 0.03	0.15 ± 0.01
Na_2_O, [wt %]	0.14 ± 0.03	0.24 ± 0.07	0.21 ± 0.03	0.88 ± 0.01	0.33 ± 0.02	0.18 ± 0.26^c^	1.30 ± 0.02	0.16 ± 0.01
K_2_O, [wt %]	34.67 ± 0.76	27.65 ± 0.6	10.77 ± 0.37	61.42 ± 0.1	6.83 ± 0.08	2.99 ± 1.05	62.86 ± 0.1	1.59 ± 0.04

a Mean value and standard deviation for two measurements.

b Value and absolute error of single measurement.

c One of measurements was zero.

Water leaching is also more effective in removing compounds containing phosphorus from straw compared to leaching with acetic acid solution. Na and K are more completely removed from straw by acetic acid solution leaching than water leaching. This can be explained by the fact that acetic acid solution not only leaches their soluble salts, but also cations H^+^ of acetic acid are partly exchanged for K^+^ and Na^+^, which are found in insoluble organic compounds and converted to soluble acetates.

Ca and Mg compounds are also better removed by acetic acid solution leaching; however, this cannot be considered positive unequivocally: on the one hand, this is positive for reducing the ash content; on the other hand, this means the removal of refractory oxides. The change in Fe_2_O_3_ and Al_2_O_3_ content of ash can be considered insignificant by both leaching with water and acetic acid solution.

Comparing the chemical composition of the dried water leachate (Table 3, column 5) and acid leachate (Table 3, column 8), shows that a more significant amount of SiO_2_ was removed from straw by leaching with water. Water-soluble silicic acid salts K_2_SiO_3_ (*t*_melt_ = 1045 °C), and Na_2_SiO_3_ (*t*_melt_ = 1088 °C) are relatively low melting, and therefore their leaching with water can be considered positive for improving the characteristics of water-leached straw ash. Due to the washing of soluble compounds, the content of SiO_2_ increased from 40.4 wt % in the mineral part of the original straw to 68.8% in the mineral part of water-leached straw and up to 84.7 wt % of straw leached with acetic acid solution.

In Table 3, data on the chemical composition of ash obtained by low-temperature (550 °C) oxidation of the studied straw samples determining the ash content according to the standard method are presented. Comparing the chemical composition of the mineral part of the original straw and its ash (columns 2 and 3), the mineral part of the water-leached straw and its ash (columns 4 and 6), and the mineral part of the straw leached with a 10% solution of acetic acid and its ash (columns 7 and 9), there was a significant decrease in the content of potassium oxide K_2_O in the ash compared to its content in the mineral part of the corresponding straw sample. This can be explained by the emission of K_2_O even at 550 °C during straw combustion (ashing, oxidation).

In addition, it should be noted that after oxidation of both original straw and leached straw samples, there was a significant decrease in the content of calcium oxide in the ash compared to its content in the mineral part of the corresponding straw sample (Table 3). CaO is a refractory oxide, and its evaporation at low temperatures should not occur. It is likely that during the straw ashing, the compounds or particles containing CaO were carried out by a mechanism we do not understand. It is worth considering the possible presence of calcium oxalate CaC_2_O_4_, which at a temperature of 400 °C begins to decompose into CaCO_3_ and CO + CO_2_ (Curetti et al.),^50^ and the emission of atomized CaCO_3_ can be assumed. It is noteworthy that even when determining the ash content with low-temperature oxidation of biomass in a muffle furnace at a temperature of 550 °C, loss of ash components can occur, as was warned in the ISO 21404:2020.^13^

Due to the partial loss of light evaporating and flying components of the ash during straw ashing, the resulting ash was enriched with SiO_2_, with an increase in its content from 40% in the mineral part of the original straw to 50% in its ash.

For water-leached straw and acetic acid solution-leached straw, an increase in the content of SiO_2_ in the ash occurred. First, due to the predominant leaching of more soluble components with an increase in the SiO_2_ content from 40% in the mineral part of the original straw to 68.8 and 84.7% in the leached straw samples; second, due to the loss of light evaporating ash components in low-temperature oxidation of the leached straw with a final increase of the SiO_2_ content to 81 and 94% in the ash of the leached straw samples.

The described results show that even at low-temperature oxidation of straw in the muffle furnace, processes occurred that led to the formation of ash, whose chemical composition differs significantly from the composition of the mineral part of straw. In this regard, according to the data on the ash element content in biomass, which were determined according to standards ISO 16967:2011^51^ and ISO 16995:2015,^52^ it is not possible to unambiguously predict the composition of ash that can be obtained after straw burning.

According to standard ISO 16967:2011,^51^ there are two procedures for determining the content of major ash-related elements: Al, Ca, Fe, Mg, P, K, Si, Na, and Ti (with recalculation to oxide content) directly in the fuel or by their content in ash. At the same time, the standard does not note the possible difference in the results according to these two procedures. The data stated above showed the difference in the content of ash-related elements in the straw and in the ash obtained from it. Therefore, it is necessary to investigate the straw leaching with the determination of ash-related components in the straw and in the resulting ash, which are not identical.

### Heavy Metal Content in Straw Samples

Data on the measured contents of the heavy metals in original and leached straw are presented in Table 4. There are limitations on the contents of eight metals, and seven of them were found in the studied straw. The contamination of the original straw with Cr exceeded the limitation, which was not removed by leaching; instead, it was accumulated, which resulted in the content increase. Cr content was in known range of contamination in the original straw, in accordance with the range mentioned in ISO 17225-1,^4^ and one can suppose that wheat grew on contaminated land. The contents of other heavy metals were below limitations, and the straw leaching allowed the contents to slightly decrease. Because of its high Cr content, the studied straw, both in the original and leached states, cannot be suitable for the production of nonindustrial straw pellets.

**Table 4:** Heavy Metal Content in Straw Biomass^a^

heavy metals	limitations of ISO 17225-6:2014^5^ on heavy metals content in straw pellets	original straw	straw leached with water	straw leached with 10% solution of acetic acid
As, [mg/kg]	1	<0.3	<0.2	<0.1
Cd, [mg/kg]	0.5	<0.3	<0.2	<0.1
Cr, [mg/kg]	50	59.3	73.1	67.1
Cu, [mg/kg]	20	8.5	5.0	4.6
Pb, [mg/kg]	10	<0.3	<0.2	<0.2
Hg, [mg/kg]	0.1	NDS	NDS	NDS
Ni, [mg/kg]	10	3.6	2.3	1.8
Zn, [mg/kg]	100	1.8	1.0	0.5

a NDS – not detected in straw.

Leached straw unsuitable for the production of nonindustrial pellets because of its heavy metal content, due to low ash content and high temperature of ash melting, can be successfully used for industrial pellet production for usage in large and medium-scale energy installations equipped with flue gas cleaning systems which provide an appropriate reduction of emissions to levels established in EU Directives for large^53^ and medium^55^ combustion plants, and for other industrial installations.^54^

### Results of TGA-DSC of Straw Ash

**Figure 2:**
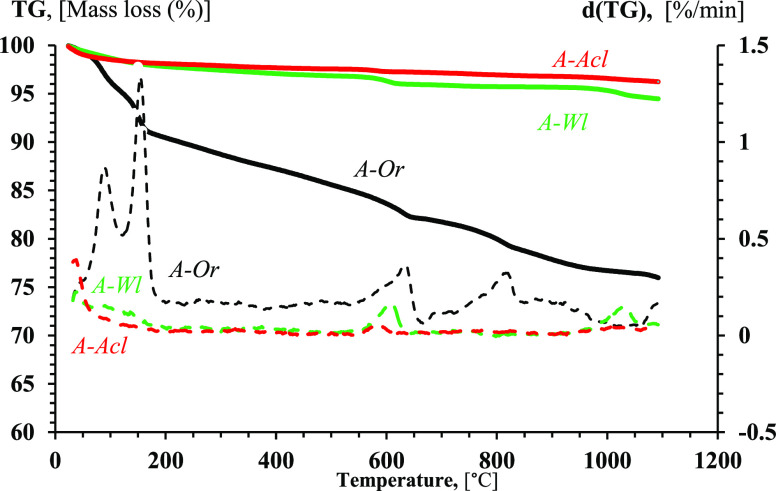
TGA (solid) and d(TG) (dashed) curves for ashes of original straw (A-Or), water-leached straw (A-Wl), and 10% acetic acid solution-leached straw (A-Acl) at an air flow rate of 100 mL/min and a heating rate of 10 °C/min.

**Figure 3:**
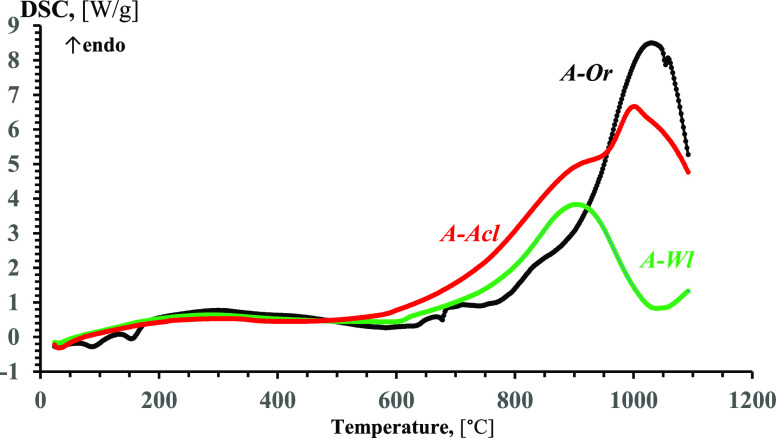
DSC curves for ashes of original straw (A-Or), water-leached straw (A-Wl), and 10% acetic acid solution-leached straw (A-Acl) at an air flow rate of 100 mL/min and a heating rate of 10 °C/min.

TGA-DSC curves for the ashes of the original and leached straw samples are shown in Figures 2 and 3. Results were interpreted according to general approaches stated in paper^40^ taking into consideration experience of studying bottom and fly ash of straw burned in boiler^56^ and characterizing laboratory straw ash.^57^ For ash of original straw (A-Or) in the temperature range of 20–550 °C, the d(TG) curve shows two peaks, at 100 and 150 °C, corresponding to evaporation of 9 wt % of capillary and colloidal water with small endothermic peaks on the heat flow curve. In the temperature range of 180–550 °C, there was an endothermic process of crystal hydrate decomposition with water evaporation of 6 wt %.

For the ash of water-leached straw (A-Wl), the TG curve shows 2 wt % evaporation of capillary water at 20–60 °C with a peak rate at 38 °C on the d(TG) curve, and small evaporation of colloidal water in the range of 60–170 °C with a blurred peak at d(TG) curve. In the temperature range of 170–550 °C, there was an endothermic process of crystal hydrate decomposition with water evaporation of 1.5 wt %.

For ash of acetic acid solution-leached straw (A-Acl), the TG curve shows evaporation of mainly capillary water at 20–60 °C with a peak rate at 38 °C on d(TG) curve, and there can be marked small evaporation of colloidal water in the range of 60–170 °C. In the temperature range of 170–550 °C, there was an endothermic process of crystal hydrate decomposition with water evaporation of 0.6 wt %.

These ash samples had already been calcined for a long time (at least 3 h) to a constant mass at a temperature of 550 °C during straw sample ashing. Therefore, the phenomena occurring during the reheating of the ash at TGA-DSC in a temperature range of 20–550 °C can be considered to be caused by water vapor absorption during the ash storage before study. The ash of the original straw (A-Or) was the most hygroscopic and absorbed during storage 15 wt % of moisture, the ash of water-leached straw (A-Wl) was less hygroscopic and absorbed 3.5 wt % of moisture, and the least hygroscopic was the ash of acetic acid solution-leached straw (A-Acl), which absorbed only 2.4 wt % of moisture. This correlates with the alkali metal oxide content of the corresponding ash samples. It can be assumed that pellets made of original straw will intensively absorb moisture and can loosen and crumble when stored. On the contrary, it can be predicted that pellets made of leached straw will absorb moisture to a lesser extent, and one can expect their better stability at storage.

In the temperature range of 550–660 °C, there was decomposition of carbonates, showing mass decrease of 2.5 wt % for the ash of original straw (A-Or) due to CO_2_ release on TG curve, with a distinct peak at 630 °C on the d(TG) curve with slight rise of endothermic heat flow at DSC curve. In this temperature range, the TG curve of water-leached straw ash (A-Wl) shows a 0.75 wt % mass decrease with a distinct peak of mass decrease rate at 604 °C on the d(TG) curve. For acetic acid solution-leached straw ash (A-Acl), there was 0.25 wt % mass decrease at the TG curve and a small peak of mass decrease rate at 585 °C on the d(TG). The content of carbonates of alkali and alkali-earth metals in the ash samples can be characterized by the mass of CO_2_ released at TGA-DSC. The total content of the alkali and alkali-earth metal oxides in the ash samples correlates with a mass decrease in the temperature range of 550–650 °C found at TGA-DSC tests. A significant decrease in CO_2_ release is indirect evidence of the alkali and alkali-earth metal removal at straw leaching.

For A-Or in the temperature range of 660–750 °C, there was 1 wt % mass decrease due to the evaporation of ash components, presumably K_2_O. At 751–835 °C, there was a drastic rise of heat flow with 2.1 wt % mass decrease at TG and peak at d(TG) at 815 °C which can be interpreted as melting of salts mixture CaCl_2_·KCl·NaCl·KPO_3_ with the evaporation of ash components, presumably of KCl as having among named salts the largest vapor pressure at this temperature interval. From DSC curve follows the onset of ash melting for ash of original straw at about 740 °C.

For A-Or from 835 °C and up to 1100 °C, there was a mass decrease at TG with a peak at the DSC curve at 1028 °C, which can be attributed to the melting of ash, presumably sulfate Na_2_SO_4_, silicates K_2_SiO_3_ and Na_2_SiO_3_, and then continued with the melting of complex mixture of oxides available in ash, with evaporation/emission of 3 wt % of ash components with a slowing of mass decrease rate as ash components easy for evaporating are being exhausting.

In the temperature range from 660 up to 920 °C for A-Wl and A-Acl, there were no remarkable changes in mass, and this can be explained by the very low content of K_2_O and Cl in these ashes. In this temperature range, ashes of leached straw undergo endothermic transformations with considerable energy demand. From DSC curves, it can be assumed that the onset of melting is at 750 °C for A-Wl, and at about 800 °C for A-Acl.

For A-Wl from 920 to 1100 °C, there was 1 wt % mass decrease at TG with peak of mass decrease rate at 1026 °C on the d(TG) curve with slowing and rising of heat flux, which can testify about evaporation/emission of ash components and transition to fusion of high-melting components of ash.

For A-Acl at 920–1100 °C, there was 0.5 wt % mass decrease at TG with local peak of mass decrease rate at 1020 °C on the d(TG) curve with peak of heat flux at 1002 °C. This can also testify the transition to fusion of high-melting ash components.

Because of instrument limitation for heating only to 1100 °C, TGA-DSC experiments were not finished to full melting of ash samples; nevertheless, TGA-DSC curves illustrated change in ash behavior at thermal analysis due to straw leaching. Comparing the found onsets of ash sample melting with data on their IDTs described below, one can see that the onset of melting began far before reaching IDTs.

### Results of Characteristic Temperatures of Ash Melting Behavior Measurements

In experiments, the IDT for the ash of the original straw was identified by the swelling of the test pyramid or its apex inclination for an angle of more than 20–30° from the vertical position. Individual values of IDT were in the range of 830–863 °C, and as a result, the average value of IDT = 847 °C was taken. When heating continued to 990 °C, a significant decrease in size and deformation of the shape of the pyramids were noted but the formation of a spherical surface was not observed.

The determination of the IDT for the ash of leached straw samples differed from that of the ash of the original straw. In the first experiments at furnace temperatures up to 1100 °C, no signs of deformation were noted, the experiment was stopped, and after cooling, samples were examined and no changes in shape or size were found. The pyramids were solid and destroyed with considerable force, which indicated their sintering.

When a sample of A-Wl was heated to 1250 °C, a slight rounding of the sharp apex of the pyramid was observed. After the experiment, it was revealed that the rounded apex of the pyramid became transparent, and this also confirms the beginning of ash melting.

When heated to the maximum possible temperature of 1275 °C, pyramids of A-Acl showed no changes in their shape or size, and therefore IDT > 1275 °C was accepted.

### Ternary Diagram

**Figure 4:**
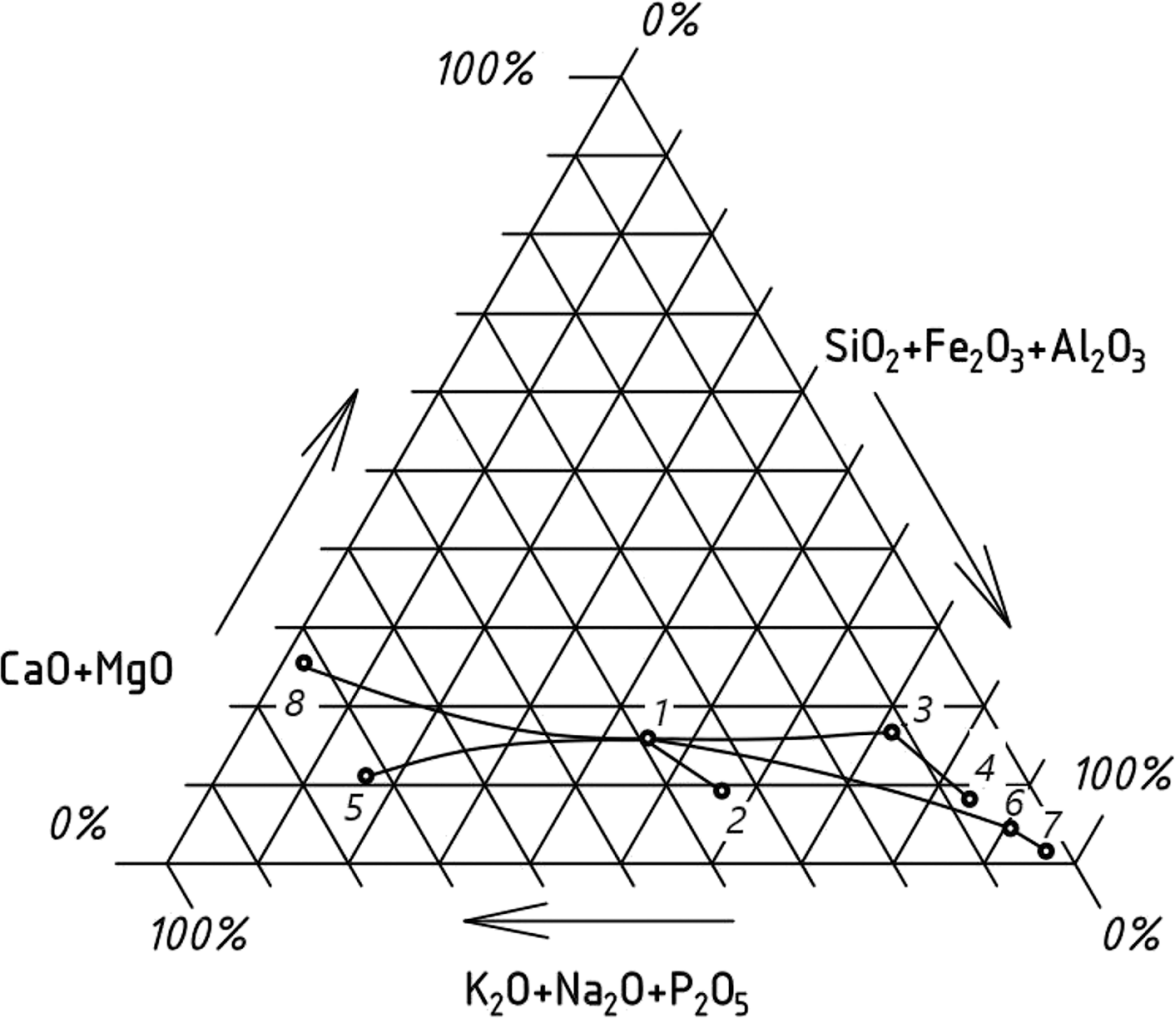
Ternary diagram of ash-related components and ash of original and leached straw: *states:* (1) ash-related components in original straw; (2) ash of original straw; (3) ash-related components in water-leached straw; (4) ash of water-leached straw; (5) ash-related components in dried leachate of water leaching; (6) ash-related components in straw leached with acetic acid solution; (7) ash of straw leached with acetic acid solution; (8) ash-related components in dried leachate of leaching with acetic acid solution; processes: (1–2) ashing at 550 °C of original straw; (1–3) straw leaching with water; (1–5) ash-related component removal at water leaching of straw; (3–4) ashing at 550 °C of water-leached straw; (1–6) straw leaching with acetic acid solution; (1–8) ash-related components removal at straw leaching with acetic acid solution; and (6–7) ashing at 550 °C of acetic acid solution-leached straw.

A ternary diagram with the pointed-out composition of ash-related components in original and leached straw samples, as well as the ash composition of original and leached straw samples and the composition of ash-related components removed with leachates, is presented in Figure 4. Also, there are lines between points reflecting processes that occurred. As one can see from the diagram, leaching of original straw with water (process 1–3) and leaching of original straw with acetic acid solution (process 1–6) caused shifting of the composition of ash-related oxides in straw to the right bottom cone, enriched with high-melting components SiO_2_, Al_2_O_3_ and Fe_2_O_3_ (the last is high melting in an oxidative environment) and depleted with low melting components K_2_O, Na_2_O, and P_2_O_5_.

Ashing of straw samples at 550 °C in a muffle furnace caused shifting from the state of ash-related component composition in straw to the composition of the obtained ash: oxidation of the original straw (process 1–2), water-leached straw (process 3–4), and acetic acid solution-leached straw (process 6–7). Straw sample oxidation also occured with shifting the ash composition to the right bottom cone, to states with a higher content of high-melting components and a lower content of low melting components.

Straw leaching was associated with the removal of soluble ash-related components from straw with water leaching (processes 1–5) and with acetic acid solution leaching (process 1–8). Dried leachate of water leaching (point 5) was considerably enriched with low melting components (K_2_O + Na_2_O + P_2_O_5_) and slightly depleted with (CaO + MgO) compared to those of original straw. Dried leachate of acetic acid solution leaching (point 8) was also considerably enriched with low melting components (K_2_O + Na_2_O + P_2_O_5_) and slightly enriched with (CaO + MgO) compared to those of original straw and leachate of water leaching.

It should be mentioned that the ashes of all straw samples remained in the third region of the high slagging and fouling propensity of Lachman’s ternary diagram.^48^ It means that there is a need to improve Lachman’s diagram by separating a zone for leached agricultural biomass.

From the phase diagram for the system of CaO·K_2_O·SiO_2_ elaborated by Roedder,^58^ it is evident that for straw ash containing about 9% CaO, shifting its composition to a zone of 80%<SiO_2_ < 88% means transition from zones of low melting eutectics to zone of tridymite with melting in temperature range of 1100–1500 °C, and at SiO_2_ > 88% to zone of cristobalite with melting above 1500 °C. As to composition, A-Wl (SiO_2_ = 81.4%) falls into zone of high-melting tridymite, and the A-Acl (SiO_2_ = 94.3%) falls into the zone of refractory cristobalite. This conclusion correlates with our results from the IDT measurements.

### Ash Melting and Fouling Indexes

Slagging and fouling indexes, which are used to characterize some aspects of ash melting and associated problems of studied straw samples, are listed in Table 5.

**Table 5:** Slagging and Fouling Indexes and Inclinations to Slagging and Fouling for Studied Ashes of Original and Leached Straw Samples

indexes	ash of original straw	ash of water-leached straw	ash of straw leached with 10% acetic acid	critical values and slagging and fouling inclinations
silica content, SiO_2_, [wt %] in ash	50	81	94	
	high	high	high	<20 low slagging;^59^ 20–25 medium;^59^ >25 high^59^
	high	low	low	**our proposal:** >80 low slagging; < 80 high
chlorine content, Cl, [wt %] in dry biomass	0.216	0.038	0.058	<0.2 low slagging and fouling;^49^ 0.2–0.33 medium;^49^ >0.5 high^49^
	medium	low	low	
basic to acidic compounds ratio, B/A, -	0.8	0.2	0.1	<0.5 low slagging;^48^ 0.5–1.0 medium;^48^ 1–1.75 high;^48^ >1.75 severe^48^
	medium	low	low	
bed agglomeration index, BAI	0.006	0.032	0.106	<0.15 high agglomeration^48^
	high	high	high	
fouling index, Fu, [wt %] in ash	21.9	1.5	0.1	<0.6 low fouling;^49^ 0.6–40 medium;^49^ >40 high^49^
	medium	medium	low	
slag viscosity index, Sr, [wt %] in ash	85	91	98	>72 low slagging;^48^ 65–72 medium;^48^ <65 high^48^
	low	low	low	
IDT, [°C]	847	1250	>1275	>1100 low slagging;^59^ 900–1100 medium;^59^ <900 high^59^
	high	low	low	

According to critical values for silica content which were stated in paper,^59^ ashes of original and leached straw having silica content above 25 wt % pose a high inclination to slagging and fouling. It seems that such a critical value is not suitable to characterize the slagging and fouling of leached straw, and a special study is necessary to establish an appropriate critical value for silica content. Preliminarily, based on the data of Jenkins et al.^11^ and on our above-described analysis of ternary diagram of leached straw ash, the critical value of silica content in straw ash SiO_2_ = 80% can be proposed: at SiO_2_ > 80%, the low slagging and fouling inclination, and at SiO_2_ < 80%, high inclination, without range of medium inclination. At such a critical value, the ash of original straw has high, but ashes of leached straw have low inclination to slagging and fouling.

As to chlorine content, original straw possesses a medium inclination toward slagging and fouling, and the ash of leached straw is low. Similar characteristics of straw inclination to slagging and fouling can be concluded from the values of the basic to acidic compounds ratio, B/A.

Fouling index Fu shows low slagging and fouling inclination for A-Acl, medium for A-Wl, and medium-to-high for A-Or. The slag viscosity index shows a low inclination to slagging and fouling for ashes of both original and leached straw. Values of IDT show a high inclination toward slagging and fouling for the ash of original straw but low for the ash of leached straw.

Special attention should be paid to the bed agglomeration index (BAI), which is used to characterize ash agglomeration at fluidized bed combustion.^60^ According to the recommended critical values of BAI, both original and leached straws have a high inclination toward agglomeration. It seems that, at least for A-Acl having an IDT > 1275 °C, the application of such a critical value is doubtful. So, there is a need for additional studies dedicated to agglomeration of ash of leached straw.

## Conclusions

### Main Scientific Results of the Study

Leaching of straw, including soaking with heating to 100 °C and subsequent plug flow flashing in press with control of leaching completion in the absence of chlorine in leachate, allowed the ash content to decrease from 7.15% in the original straw to 3.93% at water leaching and to 4.29% at leaching with a 10% solution of acetic acid. The yield of the dry mass of leached straw in relation to the dry mass of the used original straw was 91.7% at water leaching and 94.8% at leaching with acetic acid solution. The liquid to biomass ratio was 0.029 l/g at water leaching and 0.031 l/g at leaching with acetic acid solution.

Due to leaching according to the proposed approach, the decrease in content of chlorine, nitrogen, and sulfur below the limitations for nonindustrial straw pellets was achieved. Straw leaching with acetic acid solution until the absence of chlorine in the leachate turned less efficient for chlorine and sulfur removal.

Due to the predominant leaching of more soluble ash-related components Na and K, Ca and Mg from straw, and due to the loss of K_2_O and CaO even at low-temperature ashing of straw, the SiO_2_ content in the ash increased from 50% in the ash of original straw to 81.4% in the ash of water-leached straw and to 94.3% in the ash of acetic acid solution-leached straw. As a result of leaching, the melting onset of the ash shifted to higher temperatures. The determined IDT for the ash of the original straw was 847 °C, and for the ash of water-leached straw, it increased to 1250 °C, and for the ash of acetic acid solution-leached straw, it increased to 1275 °C. In the ternary diagram, the ash compositions due to leaching were shifted into zones of high-melting tridymite and cristobalite.

The original straw was contaminated with Cr to 59.3 mg/kg, but straw leaching did not allow a decrease in its content. The contents of other heavy metals in the original straw were below limitations, and at leaching, they were changed insignificantly.

By TGA-DSC study, it was found that the ash of the original straw was the most hygroscopic, absorbing up to 15 wt % of moisture, and the ash of leached straw was less hygroscopic. It can be expected that pellets made from leached straw will absorb moisture to a lesser extent and will be more stable at storage.

### Results of Complex Study of Wheat Straw

The original straw was not suitable for the production of nonindustrial pellets because of the high contents of chlorine, sulfur, and Cr and low IDT of ash. Almost all indexes of straw were improved due to leaching with water or water solution of acetic acid, but Cr content was far above limitation, thus even leached straw remained unsuitable for production of nonindustrial pellets.

The practical significance of the gained results lies in the fact that monitoring the absence of chlorine in the leachate can be an acceptable control parameter for the completion of straw leaching. Before straw procurement, producers of pellets need to assess their suitability as to heavy metal content, both in the original and leached states.

Possible direction for further research is a study of Ca compound transformation at straw burning to disclose the mechanism of Ca emission leading to a decrease of its content in ash compared to its content in the mineral part of the straw.

## Abbreviations

A-Or: ash of original straw
A-Acl: ash of acetic acid solution-leached straw
A-Wl: ash of water-leached straw
B/A: basic to acidic compounds ratio
BAI: bed agglomeration index
Fu: fouling index
m toe: million tonnes of oil equivalent
MW: megawatt
NDS: not detected in straw
STD: standard deviation
Sr: slag viscosity index
TGA-DSC: simultaneous thermogravimetric analysis and differential scanning calorimetry
XRFA: X-ray fluorescence analysis
SST: shrinkage starting temperature
DT: deformation temperature
HT: hemisphere temperature
FT: flow or fluid temperature
IT (often abbreviated as IDT): initial deformation temperature
ST (often abbreviated as SOT): softening temperature
